# Quality of care should be a core consideration in health systems recovery from COVID-19

**DOI:** 10.1093/ijcoms/lyab005

**Published:** 2021-07-06

**Authors:** Matthew Neilson, Sheila Leatherman

**Affiliations:** Independent public health consultant, UK; Gillings School of Global Public Health, University of North Carolina, Chapel Hill, NC, USA

**Key Messages**

- COVID-19 has highlighted once more the need for urgent attention to the quality of health care globally.
- As health systems plan recovery from COVID-19, there will be opportunities to systematically address quality of care.
- By focusing on quality in COVID-19 recovery, health systems can not only improve population health outcomes but also improve their resilience against future crises.
- Practical steps to address quality of care in COVID-19 recovery include considering quality in formal recovery planning; strengthening national strategic direction on quality, built around a set of quality interventions; monitoring and publicly reporting on quality of care; and leveraging existing assets and expertise on quality of care.

## Abstract

Coronavirus disease-2019 (COVID-19) has shocked health systems globally, overwhelming rich and poor countries and highlighting both the need for and risks to quality care. Massive and persistent problems have been encountered in providing public health interventions as well as acute and chronic care for COVID-19 and in maintaining reliable provision of essential health services, already daunting for much of the world in so-called ordinary times. A survey from mid-2020 highlighted that 90% of the included countries had disruptions to their essential health services, with low and middle income settings worst affected [1]. The COVID-19 pandemic is expected to result in vast increases in childhood deaths, owing to compromised health services, disrupted vaccination programmes, and malnutrition, among other challenges [2]. While recorded deaths from COVID-19 have passed the grim milestone of 2.5 million in early 2021, overall excess mortality could be up to 1.6 times higher [3].

Even without the increased pressures of COVID-19, poor quality care is responsible for between 5.7 and 8.4 million deaths each year in low- and middle-income settings [4]. As countries now start to look towards recovery, it is not unreasonable to imagine the legacy of damaged health systems may cause increased morbidity and mortality from poor quality care. But there is also an opportunity to ensure efforts to ‘build back better’ take advantage of the growing base of evidence and experience on addressing quality of care.

‘Health systems recovery’ is a term used to describe the process of restoring and improving the functions of a health system that have been exposed to a shock or public health emergency, such as natural disaster, conflict, or a disease outbreak. The World Health Organization has proposed that ‘the goal of health systems recovery is to design a system that is able to respond to the demands and health needs of the population; perform its functions effectively, efficiently, and sustainably; increase health systems resilience; and mitigate the risk of future health emergencies’ [5]. The critical linkages with the quality agenda are evident, with a central aim of global efforts on quality health services being to effectively and efficiently meet population health needs [4, 6, 7]. Further, quality has been widely acknowledged as conferring a resilience advantage to health systems [8–10], acting as a foundation for effective and safe response and building the community trust critical for continued engagement and utilization during crises. Building this trust—a major challenge in many settings, especially low- and middle-income settings where less than a quarter of those surveyed believe the health system works well [7]—can be strengthened through a focus on quality of care within health systems recovery from COVID-19.

So what should a focus on quality in recovery look like? While context-specific approaches will be required, particularly in settings of extreme adversity, we propose here four main components applicable across most settings.

Firstly, there is a need for systematic consideration of quality within formal recovery planning, requiring intentional efforts to analyse quality challenges, identify opportunities for action within emerging recovery plans and programmes, and allocate adequate resource.

Secondly, recovery post-COVID-19 presents an opportunity to strengthen national strategic direction on quality, based on published global recommendations [6, 11, 12] and utilizing foundational technical resources [13, 14]. Any national strategy should incorporate a pragmatic set of quality interventions to drive change at multiple health system levels. Drawing from existing lists of evidence-based interventions [6, 15, 16], Box 1 proposes a focused set that may be of particular relevance in the context of COVID-19 recovery.

Box 1. Illustrative quality interventions to support health systems recovery

Ensure basic infrastructure and essential inputs

Assess facility capacity for delivery of safe and effective health services

Strengthen infection prevention and control

Use context-appropriate guidelines, standards, and protocols

Perform clinical audit and feedback

Provide training and supportive supervision of workforce

Use electronic/digital health technologies and programs

Formally engage and empower local communities

Optimize procurement and supply chain systems

Strengthen public performance reporting of process and outcomes measures

Thirdly, efforts to monitor health systems performance through COVID-19 response and recovery should systematically measure and publicly report quality of care data to allow problems to be identified, progress tracked, and accountability enforced.

Fourthly, those leading health systems’ recovery efforts should identify and leverage existing quality assets across health systems. In many countries, vast experience on quality of care already exists that can be of value in recovery, from facility-level expertise in areas such as infection prevention and control or quality improvement processes, through to national quality directorates with experience in diverse areas like standards development and community engagement. This workforce should be engaged and strengthened throughout health systems recovery.

## Data Availability

No new data were analyzed for this study.
